# Lutein-Fortified Plant-Based Egg Analogs Designed to Improve Eye Health: Formation, Characterization, In Vitro Digestion, and Bioaccessibility

**DOI:** 10.3390/foods12010002

**Published:** 2022-12-20

**Authors:** Giang Vu, Xiaoke Xiang, Hualu Zhou, David Julian McClements

**Affiliations:** Biopolymers and Colloids Laboratory, Department of Food Science, University of Massachusetts, Amherst, MA 01003, USA

**Keywords:** encapsulation, emulsions, INFOGEST, bioaccessibility, lipid hydrolysis

## Abstract

Lutein is a carotenoid found in real eggs that has been reported to have beneficial effects on eye health by reducing the risk of age-related macular degeneration. However, lutein is not often included in plant based (PB) egg analogs. It would, therefore, be advantageous to fortify PB eggs with this health-promoting carotenoid. Moreover, lutein is a natural pigment with a bright red to yellowish color depending on its concentration and environment. It can, therefore, also be used as a plant-based pigment to mimic the desirable appearance of egg yolk. Some of the main challenges to using lutein as a nutraceutical and pigment in PB foods are its poor water-solubility, chemical stability, and bioavailability. In this study, we encapsulated lutein in oil-in-water emulsions, which were then utilized to formulate whole egg analogs. Ribulose-1,5-bisphosphate carboxylase/oxygenase (RuBisCO) protein isolated from a sustainable plant-based source (duckweed) was used to mimic the thermally irreversible heat-set gelling properties of globular egg proteins, with the aim of obtaining a similar cookability and texture as real eggs. The lutein content (80 mg/100 g) of the egg analogs was designed to be at a level where there should be health benefits. The protein (12.5 wt.%) and oil (10 wt.%) contents of the egg analogs were selected to match those of real egg. The effects of oil droplet size and oil type on the bioaccessibility of the encapsulated lutein were examined using the INFOGEST in vitro digestion model. For the emulsions formulated with long chain triglycerides (LCTs, corn oil), lutein bioaccessibility significantly increased when the initial droplet diameter decreased from around 10 to 0.3 μm, which was attributed to more rapid and complete digestion of the lipid phase for smaller droplets. For medium chain triglycerides (MCTs), however, no impact of droplet size on lutein bioaccessibility was observed. A high lutein bioaccessibility (around 80%) could be obtained for both LCTs and MCTs emulsions containing small oil droplets. Thus, both types of oil can be good carriers for lutein. In summary, we have shown that lutein-fortified PB eggs with good digestibility and bioaccessibility can be created, which may play an important role in ensuring the health of those adopting a more plant-based diet.

## 1. Introduction

Recently, many sectors of the food industry have shifted their focus toward the development of plant-based foods to meet increasing consumer demand for this kind of product. Indeed, the Good Food Institute (GFI) reported that the total sales of plant-based food products reached roughly $7 billion in 2020, with over 800 companies targeting this market globally [1]. The main driving forces for the increase in interest in plant-based foods are consumer concerns about the negative impacts of the livestock industry on the environment, animal welfare, and human health [2]. Reducing the amount of animal-derived products, such as meat, fish, egg, and milk, in the human diet could help to mitigate the adverse effects of the livestock industry on greenhouse gas emissions, land use, water use, pollution, and biodiversity loss [3]. However, it is important that any plant-based analog of an animal-derived product should have a similar or better nutritional profile, otherwise there may be adverse health consequences of switching from a conventional omnivore to a plant-based diet.

Egg analogs are an important segment of the plant-based food market [1]. These products are sold in various formats, including powders, liquids, and ready-made products. A variety of edible biopolymers are used as functional ingredients in these products to simulate the desirable physicochemical, sensory, and nutritional attributes normally provided by the globular proteins and glycoproteins in real egg. These ingredients include proteins (like lupin, mung bean, or soy proteins) and polysaccharides (like xanthan gum and starch), which are sometimes used in combination [4]. In this study, we used ribulose-1,5-bisphosphate carboxylase-oxygenase (RuBisCO) extracted from a sustainable source (duckweed) to formulate plant-based egg analogs, since our previous study showed that this plant protein could mimic the cookability and textural attributes of real egg [5]. The thermal denaturation temperature of RuBisCO protein (66 °C) is somewhat similar to those of real egg proteins (66 and 81 °C). Moreover, the gelling temperature and final gel strength of RuBisCO protein solutions can be designed to be comparable to those of real cooked eggs by optimizing the concentration of plant protein used [5]. 

Commercial plant-based eggs are designed to simulate the desirable functional and sensory attributes of real eggs but they often do not match their desirable nutritional profiles [6]. Moreover, many of the existing products are considered to be “highly processed” because they contain a long list of unfamiliar ingredients [7]. The availability of “clean-label” plant-based egg analogs with good sensory and nutritional attributes could lead to an increase in consumer interest in this product category [6]. Moreover, there is the possibility of designing egg analogs to be healthier than real eggs. For example, they can be formulated without using proteins that cause allergies or intolerances in an appreciable segment of the population (such as ovomucoid) and they can be fortified with vitamins, minerals, dietary fibers, and nutraceuticals to enhance their health benefits [8]. 

Real egg yolks contain relatively high concentrations of lutein, which is a natural pigment and nutraceutical [9]. According to previous studies [10], one egg yolk contains roughly 290 μg of lutein, which has a higher bioavailability than lutein from vegetable sources. The color of lutein varies from red to yellow depending on its concentration, which means it can be used as a pigment in plant-based foods where these colors are desired. Lutein has also been shown to exhibit nutraceutical properties, with the ability to reduce the risk of eye disease, such as age-related macular degeneration [11,12]. Lutein accumulates in the macular region of the human eye where it screens harmful blue light waves and acts as a natural antioxidant, thereby reducing oxidative stress on the retina [13]. Studies have found that consumption of around 6–10 mg of lutein per day may have these beneficial health effects [14]. However, American adults typically only obtain around 1–2 mg of lutein per day from their diets, which is considerably below the level required to see any health benefits [15]. Consequently, there is a need to fortify foods with this health-promoting carotenoid. However, this is challenging because lutein is a hydrophobic and chemically reactive molecule, which has a low water-solubility, chemical stability, and bioavailability [16,17]. Consequently, there has been great interest in utilizing encapsulation technologies to increase the dispersibility, stability, and bioavailability of lutein [17,18]. Many kinds of encapsulation technologies have been developed for this purpose, including microemulsions, emulsions, liposomes, and biopolymer particles [19,20]. However, emulsion-based systems are one of the most widely used because they are simple to formulate with common food grade ingredients and they can be economically produced at a large-scale using common food processing operations [20,21]. In vitro digestion experiments have shown that encapsulation of lutein in emulsions can greatly increase its bioaccessibility [19,22], whereas in vivo animal feeding experiments have shown it can also increase its oral bioavailability [23]. 

In this study, we used lutein-loaded emulsion-based delivery systems to fortify plant-based egg analogs with lutein. The egg analogs were designed to have a similar protein and fat content as real eggs. The level of lutein fortification was selected based on the concentrations shown to have an efficacious health effect. The impact of emulsion formulation (oil type and droplet size) on the stability and bioaccessibility of the encapsulated lutein was measured under simulated gastrointestinal conditions using an in vitro digestion model, i.e., the standardized INFOGEST method [24]. We also characterized the color stability of the lutein during the thermal processing steps required to produce the cooked egg analogs. Our study should, therefore, provide valuable insights into the development of more nutritious plant-based foods. 

## 2. Materials and Methods

### 2.1. Materials

RuBisCO protein isolate (RPI) was kindly provided by Plantible Foods, Inc. (San Diego, CA, USA) with a reported protein purity of around 82%. Lutein powder was provided by LycoRed Ltd. (Be’er Sheva, Israel). Medium chain triglyceride (MCT) oil (Miglyol 812 N) was obtained from IOI Oleo GmbH (Witten, Germany). LCT (Mazola Corn oil) was purchased from a local supermarket. All the chemicals and enzymes used in the digestion experiments were purchased from the Sigma-Aldrich company (St. Louis, MO, USA) as described previously [25]. The organic solvents used in this study were analytical or HPLC grade. Double distilled water was used for the preparation of all solutions and emulsions.

#### Sample Preparation

*Protein solution:* The plant-based egg analog solution was prepared by mixing 12.5 wt% RuBisCO protein powder in 25 mL of distilled water and then storing overnight to fully hydrate and dissolve the protein. This solution was then adjusted to pH 7.0 using NaOH solution.

*Lutein-loaded oil phase:* To achieve an amount of 20 mg lutein in 25 g of final product, lutein powder was first mixed with oil phase of either medium chain triglycerides (MCT) or long chain triglycerides (LCT—corn oil). A relatively high concentration of lutein was used to facilitate its detection and to reach the level recommended to promote eye health. The oil phase was then mixed, heated (60 °C), and sonicated to disperse and dissolve the lutein in the oil.

*Coarse emulsion preparation:* The protein solution and lutein-loaded oil phase were then blended together using a high shear mixer for 4 min at the highest speed setting (Blender model: M133/1281-0, Biospec Products, Inc., ESGC, Switzerland). The resulting mixture was then heated in a water bath at 90 °C for 30 min to gel the protein and form a cooked plant-based egg analog.

*Fine emulsion preparation:* The coarse emulsion solution was first formulated. The droplet size was then reduced by sonicating for one minute using a pulse sequence that consisted of 40% power, 2 s on then 2 s off (FB505, Fisher Scientific, Madison, WI, USA). The emulsion formed was then heated in a water bath at 90 °C for 30 min to gel the protein and form a cooked plant-based egg analog.

### 2.2. In Vitro Digestion

The gastrointestinal fate of the lutein-fortified plant-based eggs was monitored using the standardized INFOGEST in vitro digestion method [24], with slight modifications. Initially, we prepared 1.25-fold electrolyte stock solutions for the oral, stomach, and small intestine phases, and pre-weighed the enzymes and other constituents required for each of these phases. HCl and NaCl were used instead of NaHCO_3_ to control the pH and ionic strength because the latter salt interfered with the pH-Stat method [24]. Our experiments were run in open-top containers and if NaHCO_3_ was used some acidification occurred during storage. All the experiments were carried out in a water bath at 37 °C, using preheated solutions [26].

*Oral phase*: 5 g of cooked sample were transferred into a beaker, mixed with 5 mL of simulated saliva fluid (SSF), which consisted of inorganic salts, calcium, and mucin, and then manually disrupted to simulate the masticating behavior of the mouth. This was achieved by compressing the egg analog samples with a spoon for 30 s before adding the simulated oral phase fluids. In this stage, the amylase activity was 75 U/mL and the mucin concentration was 1.5 mg/mL, as specified in the INFOGEST method.

*Stomach phase*: Around 10 mL of sample from the oral phase were then added to 8.6 mL of simulated gastric fluids (SGF). The mixture was then adjusted to pH 3.0 using HCl solution. Then, 0.4 mL of distilled water was added, followed by 1 mL of pepsin solution, which led to a final total volume of 20 mL. The enzymes were kept frozen and thawed before use. The activity of pepsin during this stage was 2000 U/mL. The pH of the stomach phase was maintained constant at pH 3.0 using a pH state titrator (Metrohm, USA Inc., Riverview, FL, USA) for a 2-h period by constantly stirring while titrating in HCl 0.05 M solution. The amount of HCl solution added was recorded to provide insights into protein digestion in the gastric phase.

*Small intestine phase:* 20 mL of sample from the stomach phase was then collected, then 8 mL of simulated intestinal fluids (SIF) was added, and the mixture was adjusted to pH 7.0 using NaOH solution. Then, 3 mL of bile salt solution, 40 μL of calcium chloride solution, and around 4 mL of distilled water were added sequentially. Finally, 5 mL of lipase/pancreatin solution was added to achieve a final volume of 40 mL. The final bile salt and calcium concentration in the small intestine phase were 10 mM and 0.6 mM, respectively, and the trypsin and lipase activity were 100 and 2000 U/mL, respectively. A pH stat titrator (Metrohm, USA Inc., Riverview, FL, USA) was used to record the total volume of NaOH required to keep the pH constant at 7.0 for 2 h with constant stirring.

### 2.3. Lipid Hydrolysis

The degree of lipid hydrolysis was determined as the fraction of free fatty acids (FFAs) released in the small intestine phase. A modified solvent-extraction/titration method was used to determine the FFAs released from the samples [27]. The FFA were first extracted from the samples using Folch’s method using a chloroform/alcohol mixture as a solvent [28]. Briefly, 5 mL of chloroform: methanol (2:1 *v*/*v*) solution was mixed with 2 mL of digested samples in a test tube. The whole solution was then centrifuged at 4000× *g* for 3 min until two clear separate phases were observed. The lower phase, where the FFAs were located, was then collected, and added to 100 mL of ether/ethanol (1:1 *v*/*v*) with 3 drops of phenolphthalein as an indicator for the subsequent titration step. Then, 0.01 M NaOH solution was gradually added until the solution reached its endpoint (color change). The volume of NaOH consumed was recorded and the FFAs released was calculated based on the following equation:

$$\mathrm{FFA}\left(\%\right)=\frac{V_{\mathrm{NaOH}}C_{\mathrm{NaOH}}M_{\mathrm{lipid}}}{2W_{\mathrm{egg}}}$$

Here, *V*_NaOH_ is the volume of NaOH solution required to reach the endpoint, *C*_NaOH_ (0.01 M) is the molarity of the NaOH solution used, *W*_egg_ is the weight of the plant-based egg samples, and *M*_lipid_ is the molar mass of the oil phase. The molar mass of the MCTs and corn oil were taken to be 408.6 and 824 g/mol, respectively [26].

### 2.4. Characterization of Physicochemical Properties

#### 2.4.1. Color

The CIE tristimulus color coordinates of the plant-based egg samples were measured before and after a heat treatment using a colorimeter (ColorFlex EZ 45/0-LAV, Hunter Associates Laboratory Inc., Reston, VA, USA). Standardized black and white plates with 10° detection angle were used to calibrate the device. The L* (black/white), a* (green/red), and b* (blue/yellow) values were calculated from the resulting reflectance spectra by the instrument. The total color difference (ΔE) was also calculated to provide insights into the overall change in the optical properties of the samples:

$$\Delta E=\sqrt{{\left(L*-{L*}_{i}\right)}^{2}+{\left(a*-{a*}_{i}\right)}^{2}+{\left(b*-{b*}_{i}\right)}^{2}}$$

Here, L*_i_, a*_i_, b*_i_ are the color coordinates of the plant-based egg solution before heating, and L*, a*, b* are their color coordinates after heating. In addition, the chroma difference (C*) of the samples was calculated to provide more insights into the change in their overall color intensity [29]:

$$\Delta E=\sqrt{{\left(a*-{a*}_{i}\right)}^{2}+{\left(b*-{b*}_{i}\right)}^{2}}$$

#### 2.4.2. Zeta Potential and Size Distribution

A laser diffraction instrument (Mastersizer 2000, Malvern Instruments, Worcestershire, United Kingdom) was used to determine the mean particle diameters (d_32_ and d_43_) and particle size distribution of the egg analogs after being exposed to the different stages of the gastrointestinal tract. A particle electrophoresis instrument (NanoZS, Malvern Instruments) was used to measure the zeta-potential of the particles in the egg analogs after each gastrointestinal stage. Before each measurement, the samples were diluted into the readable range of the device using an appropriate buffer solution (pH 3 for gastric phase samples and pH 7 for mouth and small intestinal phase samples).

#### 2.4.3. Microstructure

The microstructures of the samples after exposure to different regions of the simulated gastrointestinal tract were recorded using a confocal fluorescence microscopy instrument (Nikon D-Eclipse C1 80i, Nikon, Melville, NY, USA). Different components in the images were distinguished by adding 20 μL of Nile red to stain the fat and 20 μL of FITC to stain the protein to 300 μL of the samples. A 40× objective lens was used to observe the samples.

### 2.5. Lutein Bioaccessibility and Stability

After the egg analogs had been passed through the entire gastrointestinal model (mouth, stomach, and small intestine) the lutein bioaccessibility was measured. This was achieved by collecting 20 mL of digesta from the end of the small intestine phase and then centrifuging it at 15,000 rpm for 15 min at 25 °C. This caused the samples to separate into two layers: a clear upper supernatant layer and a turbid lower sediment layer. The top layer was assumed to contain the mixed micelles that solubilized any lutein released from the egg analogs.

The mixed micelle fraction was then collected and mixed with ethanol: acetone (1:1 *v*/*v*) at a 1:3 volume ratio. The resulting sample was then centrifuged at 3000 rpm for 4 min at ambient temperature. The organic fraction formed after centrifugation was yellow and transparent, indicating that lutein was dissolved within it. We then used a UV-visible spectrophotometer (Genesys 150, Thermos Fisher Scientific, Madison, WI, USA) to measure the absorbance of the organic fraction at 460 nm to determine the lutein concentration. The bioaccessibility was then calculated using the following formula:

$$\mathrm{Bioaccessibility}\left(\%\right)=\frac{C_{\mathrm{Micelle}}}{C_{\mathrm{Digesta}}}*100$$

Here, *C*_Micelle_ and *C*_Digesta_ are the concentrations of lutein in the micelle phase and the total digesta phase calculated from a standard curve (r^2^ = 0.999), respectively.

Furthermore, the stability of the lutein after passage through the gastrointestinal tract was calculated using the following formula:

$$\mathrm{Stability}\left(\%\right)=\frac{C_{\mathrm{Digesta}}}{C_{\mathrm{Initial}}}*100$$

Here, *C*_Initial_ is the concentration of lutein initially added to the egg analogs (taking into account dilution effects). In our case, we used 20 mg of lutein per 25 g of samples, which means the initial lutein concentration was 800 mg/L.

### 2.6. Statistical Analysis

All experiments were repeated twice and three measurements were made on each individual sample. The standard deviation and mean values were then calculated from these four values. ANOVA (post-hoc Tukey HSD test) software was used to determine whether there was a significant difference between the mean values of different samples (*p* < 0.05).

## 3. Results and Discussion

### 3.1. Impact of Droplet Characteristics on Appearance

Initially, we examined the impact of oil droplet characteristics on the appearances of the emulsions used to formulate the egg analogs. A similar lutein concentration was used for all samples (20 mg lutein/25 g samples). This level was chosen so that it would be sufficient to provide beneficial health effects based on previous reports [14]. The tristimulus color coordinates of the egg analogs were measured using a colorimeter before and after the samples were heated to induce a sol-gel transition to assess the color stability of the lutein. The impacts of different oil types and droplet sizes on the appearance of the emulsions were also investigated. The results of these studies are summarized in Table 1.

The coarse and fine emulsions had mean droplet diameters of around 10 and 0.3 μm, respectively, which was due to the different energy intensities used to produce them, i.e., shearing or sonication. Most of the emulsions had roughly similar color coordinates with relatively high L* values (around 60), moderate a* values (around +10), and high b* values (around +30), which indicated that they had a yellowish-reddish color (Table 1). However, the optical properties of the emulsions did depend on oil type and droplet size. Notably, the lightness of the uncooked coarse MCT emulsion was much lower than that of all the other emulsions. The origin of this effect is unknown, but it may have been due to some phase separation of the emulsions within the measurement cell of the colorimeter.

For all samples, there was a decrease in the a* and b* values of the emulsions after cooking, which suggests there was some color fading of the lutein, presumably due to its chemical degradation when exposed to high temperatures. Indeed, previous studies have reported that carotenoids undergo degradation reactions such as oxidation and isomerization when heated, which results in color fading [16,30,31]. The thermal degradation of the lutein was also reflected in the changes in the total color difference (**∆**E) and chroma (**∆**C*) of the emulsions caused by cooking (Table 1). Even so, all the samples still had an egg-like color after heating (Table 1). There were some differences in the color coordinates of the coarse and fine emulsions, with the b* values being considerably higher for the emulsions containing the smaller droplets, which suggests they had a more intense yellow color. This effect may have been due to differences in the light scattering patterns of the small and large oil droplets in the emulsions.

### 3.2. Impact of Gastrointestinal Transit on Particle Size and Charge Characteristics

**Figure 1:**
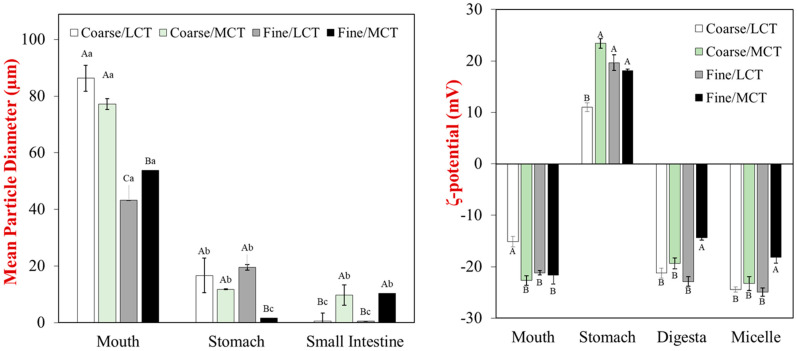
Impact of droplet size and oil type on the mean particle diameters (left) and zeta potentials (right) of egg analogs under simulated gastrointestinal tract conditions. For the particle size data, significant differences between emulsions are labeled by upper-case letters (A, B, C), whereas the significant differences among GI phases for the same sample are labeled by lower-case letters (a, b, c) with *p* < 0.05. For the particle charge data, the upper-case letters (A, B, C) show significant differences between emulsion types with *p* < 0.05. LCT: long chain triglycerides. MCT: medium chain triglycerides.

The particle size distributions, mean particle diameters (*d*_32_), and surface potentials (zeta-potential) of the particles in the different emulsions were measured after they were exposed to different stages of the simulated gastrointestinal tract (Figure 1). After exposure to the mouth phase, the mean particle diameter of all the egg analogs was much bigger than that of the initial oil droplets in the emulsions used to formulate them (Figure 1). This effect can be attributed to the fact that the egg analogs were cooked, so that appreciable protein aggregation occurred, leading to the formation of large particulates. For all emulsions, there was a substantial decrease in the mean particle diameter after they were exposed to stomach conditions. This effect can be attributed to disruption of the protein aggregates in the egg analogs due to the mechanical forces, high acidity, and protease activity of the gastric phase. There was a further reduction in the size of the particles in the egg analogs after exposure to the small intestine phase. This effect can mainly be attributed to additional disruption of the particles in the egg analogs by mechanical forces, bile salts, and the presence of proteases and lipases that digest the proteins and lipids. Interestingly, the size of the particles was larger for the emulsions containing MCTs than the ones containing LCT, which suggests that different kinds of colloidal particles were present in the digesta.

The electrical characteristics of the particles followed a similar trend for all of the egg analogs, regardless of initial droplet size or oil type (Figure 1). They had a strong negative charge in the mouth, a strong positive charge in the stomach, and then a strong negative charge in the small intestine. The high negative charge in the mouth phase can be attributed to the fact that the pH of the simulated oral fluids (pH 7) was higher than the isoelectric point of the RuBisCO proteins used to formulate the egg analogs, which has recently been reported to be around pH 5 [32]. In addition, the anionic mucin in the simulate saliva may also contribute to the negative charge measured in the mouth phase. The strong positive charge in the stomach phase can be attributed to the fact that the pH of the simulated gastric fluids (pH 3) was below the isoelectric point of the RuBisCO proteins. Presumably, the concentration of cationic protein molecules was much higher than that of the anionic mucin molecules, so the net charge was highly positive. The strong negative charge observed in the small intestine phase can again be attributed to the fact that the RuBisCO proteins were well above their isoelectric point. Moreover, the anionic bile salts and free fatty acids would also have contributed to the negative charge measured in the small intestine phase. The zeta-potential of the whole digesta was fairly similar to that of the micelle phase, which suggests that they contained fairly similar anionic species, like micelles, vesicles, and soluble protein aggregates.

**Figure 2:**
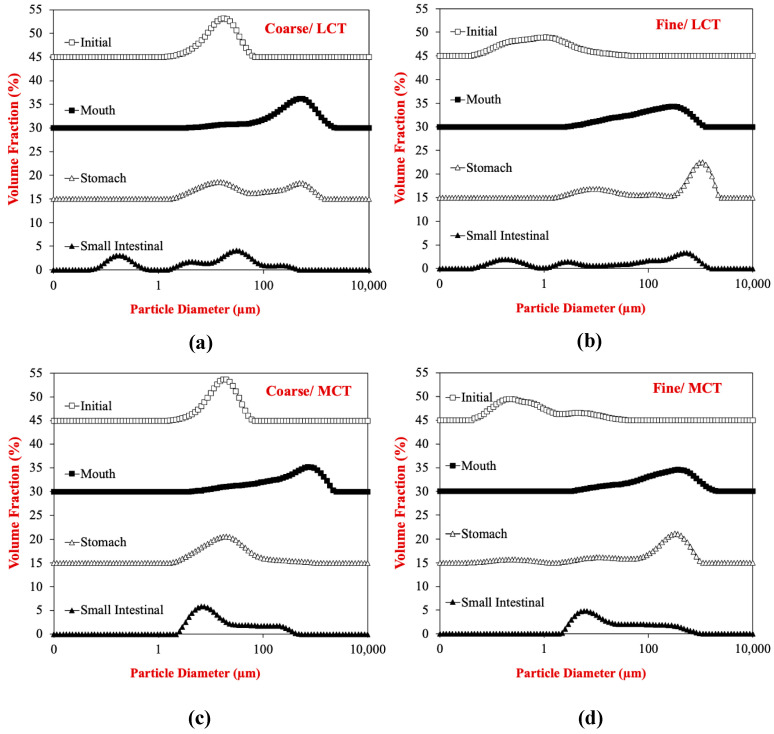
Impact of oil type (LCT or MCT) and droplet size on the particle size distributions (Coarse or fine) of egg analogs after exposure to different stages of a simulated gastrointestinal tract: Coarse/LCTs (**a**); Coarse/MCTs (**b**); Fine/LCTs (**c**); Fine/MCTs (**d**). LCT: long chain triglycerides. MCT: medium chain triglycerides. LCT: long chain triglycerides. MCT: medium chain triglycerides.

The particle size distributions obtained from laser diffraction analysis indicated that the egg analogs contained a broad range of particles with different sizes (Figure 2). In particular, there was a very wide range of particle sizes after the egg analogs were exposed to small intestine conditions. This effect can be attributed to the fact that the samples contained many different types of colloidal particle after digestion, including micelles, vesicles, liquid crystals, calcium soaps, undigested fats, and undigested proteins.

### 3.3. Microstructure

**Figure 3:**
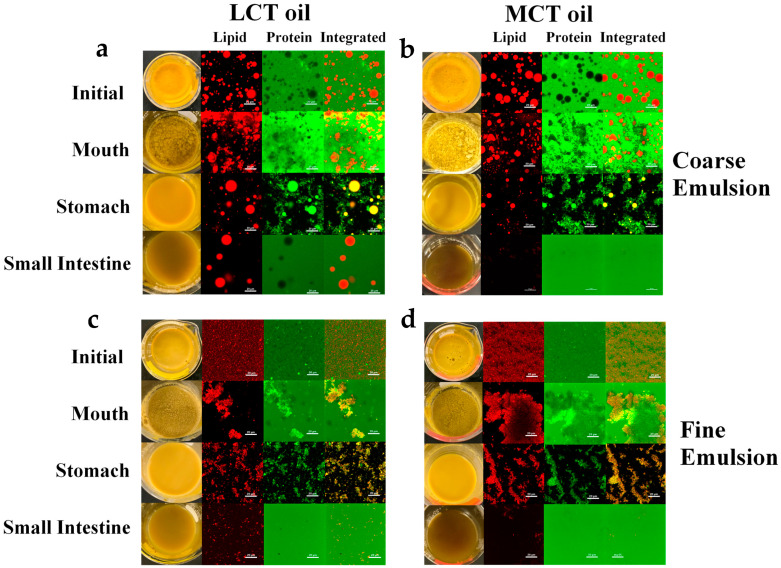
Impact of initial droplet size and oil type on the confocal fluorescence microscopy images of plant-based egg analogs after exposure to different stages of a simulated gastrointestinal tract. Lipid (red), protein (green), and integrated (both) images are presented. The white scale bars represent 20 μm. Different egg analogs represented by different letters: Coarse/LCTs (**a**); Coarse/MCTs (**b**); Fine/LCTs (**c**); and Fine/MCTs (**d**). LCT: long chain triglycerides. MCT: medium chain triglycerides.

Additional information about the structural changes in the egg analogs as they passed through the simulated gastrointestinal tract were obtained by measuring changes in their microstructure using confocal fluorescence microscopy (Figure 3). The relative locations of the lipids and proteins in these samples were ascertained using selective staining.

In Figure 3, FITC and Nile red stains were used to stain the protein (green) and lipid (red), respectively. The integrated images show the combined protein- and lipid-stained images. The images of the initial egg analogs shows that they contained oil droplets surrounded by a protein-rich aqueous phase. The fact that the proteins were initially present at the oil droplet surfaces may play an important role in lipid digestion because the proteins may have to be displaced or digested before the lipase can adsorb and hydrolyze the triacylglycerols inside the droplets. As expected, the individual oil droplets were much larger in the egg analogs prepared from the coarse emulsions than from the fine emulsions. The microstructures of the egg analogs changed appreciably as they moved through the different regions of the simulated gastrointestinal tract. In the mouth phase, the droplets in the coarse emulsions became more irregularly shaped and aggregated with each other, while the droplets in the fine emulsions became highly flocculated. In the stomach phase, the oil droplets in all the emulsions remained intact. In the coarse emulsions they appeared to exist as individual oil droplets but in the fine emulsions they existed as flocs. In contrast, there appeared to be a decrease in the amount of free protein in the aqueous phases of all the emulsions. Moreover, the proteins tended to accumulate around the oil droplet surfaces. These results can mainly be attributed to the activity of pepsin in the simulated gastric fluids, which would be expected to hydrolyze any proteins in the sample. After passing through the small intestine phase, most of the structures in the emulsions had disappeared, which can be attributed to digestion of the proteins and lipids by proteases and lipases in the simulated intestinal fluids. However, some large oil droplets did remain in the coarse LCT emulsions after exposure to the small intestine phase, which suggested that these long chain triglycerides were not fully digested by lipase. This effect can be attributed to the relatively small surface area of the oil phase exposed to the lipase when the oil droplets are large. A similar phenomenon was not observed for the egg analogs formulated from the coarse MCT emulsions because medium chain triglycerides are digested much faster than long chain ones.

### 3.4. Lipid Digestion

**Figure 4:**
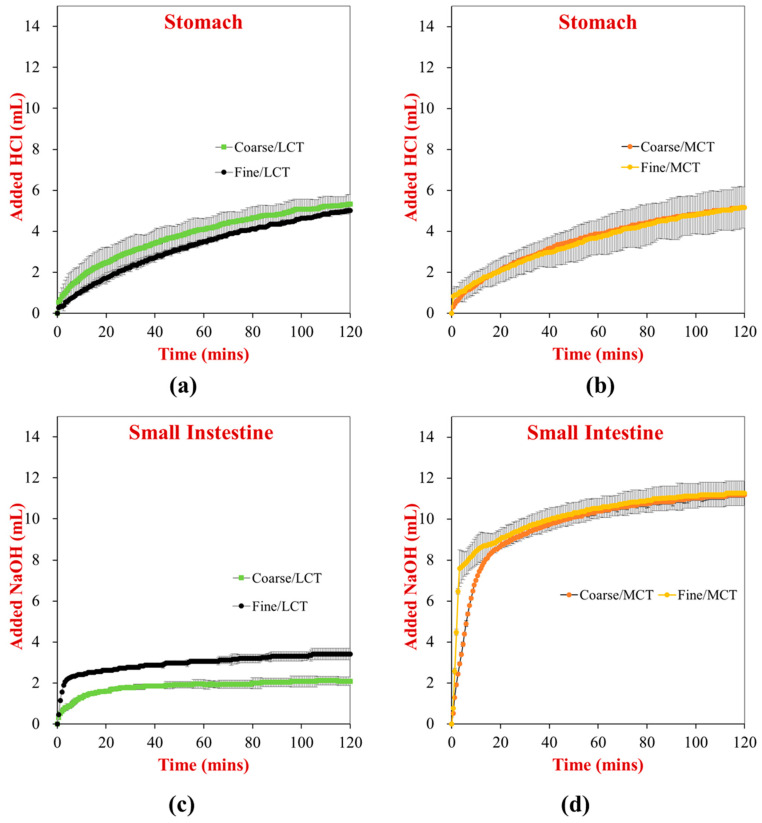
Digestion results obtained in the simulated stomach and small intestine phases using the pH stat method: LCTs in stomach phase (**a**), MCTs in stomach phase (**b**), LCTs in small intestinal phase (**c**), and MCTs in small intestinal phase (**d**). A similar vertical scale was used for all samples to obtain a more direct comparison. LCT: long chain triglycerides. MCT: medium chain triglycerides.

The digestion of the different plant-based egg analogs was monitored in the stomach and small intestine phases using the pH stat method (Figure 4). In the stomach phase (which only contained pepsin), the digestion of the proteins was followed by measuring the volume of acid solution required to keep the mixture at pH 3.0. There was no significant difference between the final volume of HCl added to the stomach phase for all samples, suggesting that protein hydrolysis was fairly similar under gastric conditions. In the small intestine phase (which contained lipases and proteases), the combined digestion of the proteins and lipids was followed by measuring the volume of alkaline solution required to keep the solution at pH 7.0. For the MCT egg analogs, the initial rate of digestion was faster for the fine emulsions than for the coarse emulsions, which can be attributed to the higher specific surface area of the fine emulsions. As a result, lipase molecules have more sites to attach to the droplet surfaces and hydrolyze the triacylglycerol molecules. Nevertheless, the final extent of lipid digestion was similar for both the fine and coarse MCT emulsions, which suggested that all of the triacylglycerol molecules had been hydrolyzed by the end of the small intestine phase. For the LCT egg analogs, the initial rate of digestion was also faster for the fine emulsions than the coarse emulsions, which can be attributed to similar reasons. However, the final extent of lipid digestion was also less in the coarse emulsions in this case, which is consistent with the confocal microscopy images. The reduced digestion of the larger oil droplets may be attributed to their smaller surface area, as well as the fact that the long-chain fatty acids generated during lipid digestion tend to accumulate at the oil-water interface. Usually, these fatty acids have to be removed by being solubilized within mixed micelles or precipitated by calcium ions. The relatively high protein concentration of the egg analogs may have inhibited these processes because the RuBisCO molecules may have bound to the micelles or calcium ions.

**Figure 5:**
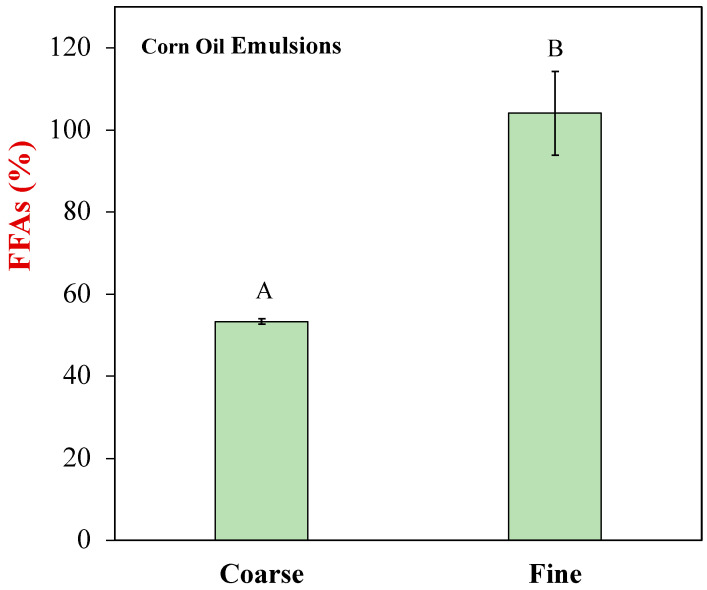
Free fatty acids released at the end point of small intestinal phase of different plant-based egg formulas. Significance difference (*p* < 0.05) of samples is labeled with capital letters (A, B).

Using pH-stat measurements, it is not possible to distinguish between lipid and protein digestion. For this reason, we used a solvent extraction method (Folch’s method) to measure the total amount of free fatty acids generated by the end of the small intestine phase for the different plant-based egg analogs (Figure 5). The MCT samples could not be analyzed using this method because the medium chain fatty acids were too polar to quantitatively remove. These results again show that the oil phase in the coarse LCT emulsions was not fully digested by the end of the small intestine phase but the one in the fine LCT emulsions was.

### 3.5. Bioaccessibility

**Figure 6:**
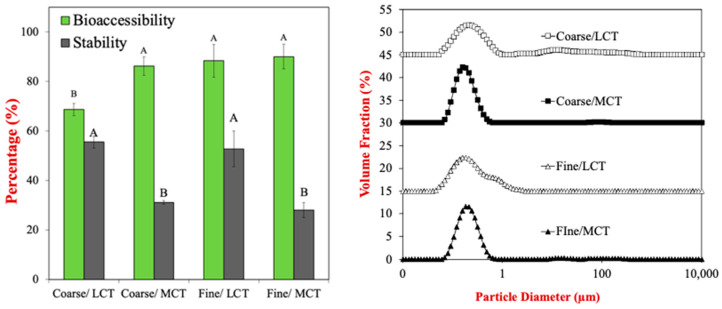
Bioaccessibility and stability of lutein (left) in different plant-based egg analogs and the particle size distribution of the micelle phase (right). Significance differences (*p* < 0.05) between samples are labeled with capital letters (A, B). LCT: long chain triglycerides. MCT: medium chain triglycerides.

The impact of initial droplet size and oil type on the bioaccessibility and stability of lutein encapsulated in the plant-based egg analogs was measured (Figure 6). These measurements were made after the egg analogs had been passed through the entire simulated gastrointestinal tract. The formation of mixed micelles occurs in the small intestine after lipid digestion. These colloidal structures consist of micelles, vesicles, and liquid crystals comprised of free fatty acids, monoacylglycerols, bile salts, phospholipids, and small amphiphilic peptides, which have hydrophobic domains capable of solubilizing hydrophobic bioactives [33].

Initially, the particle size distributions of the digesta formed after lipid digestion were measured using dynamic light scattering (Figure 6). The effectiveness of removing any large colloidal particles from the mixed micelle phase by centrifugation and filtering was highlighted by the fact that the particle size distributions of all the samples only contained a single peak between about 0.01 and 0.1 μm. The similarity in the particle size distributions of all the samples suggests that the nature of the micelles and vesicles formed were fairly similar after lipid digestion.

The bioaccessibility of the samples reflects the fraction of the lutein located in the mixed micelles after digestion. The bioaccessibility of the lutein was significantly lower in the egg analogs prepared using the coarse LCT emulsions than in all the other samples. This effect can be attributed to the fact that the oil phase was not fully digested for this sample. As a result, some of the lutein remained trapped inside the non-digested oil droplets. Indeed, the confocal microscopy and lipid digestion experiments showed that the lipids in this egg analog were not completely digested by the end of the small intestine phase. It is also possible that some of the lutein was bound to non-digested protein in the small intestine phase, which would reduce its bioaccessibility.

The impact of initial droplet size and oil type on the chemical stability of the lutein in the different egg analogs was also measured (Figure 6). In general, the stability of the lutein appeared to be somewhat higher in the LCT emulsions than in the MCT emulsions, with initial droplet size not having a major effect. This effect may have been because the larger hydrophobic domains in the mixed micelles formed from the LCTs were better able to protect the lutein.

## 4. Conclusions

In this study, plant-based egg analogs fortified with lutein were prepared. The lutein was included as a nutraceutical that may have health benefits by reducing the risk of macular degeneration, as well as a pigment for producing a yellow color like that of real whole eggs. RuBisCO protein extracted from duckweed was used as a sustainable source of a heat-set protein that could mimic the cookability and textural attributes normally provided by the globular proteins in real eggs. We showed that the gastrointestinal fate of these egg analogs depended on the initial droplet size and oil type of the emulsions used to formulate them. In particular, we showed that the best lutein bioaccessibility could be obtained using emulsions containing small oil droplets, while the best lutein stability could be obtained using emulsions formulated from LCTs. These results suggest that lutein-fortified plant-based egg analogs with the highest bioaccessibility and stability could be obtained by using LCT emulsions with small droplet sizes. Nevertheless, MCTs also seems to be suitable for preparing lutein-fortified egg analogs and it has the advantage that either small or large oil droplets can be used without affecting the bioaccessibility. In future, it will also be important to establish a better quantitative method to assess the extent of lipid and protein digestion in complex food matrices that contain both components. Finally, it will be useful to test the sensory attributes of these egg analogs, as well as their bioavailability using animal or human feeding studies.

## Figures and Tables

**Table 1:** Impact of droplet characteristics on the color coordinates of raw and cooked emulsions: L* (lightness/brightness), a* (redness/greenness), and b* (yellowness/blueness). Significant differences between raw and cooked samples are labeled by upper-case letters (A, B), whereas significant differences among oil types and droplet sizes are labeled by lower-case letters (a, b, c, d) with *p* < 0.05. The photographic images are for cooked samples. LCT: long chain triglycerides. MCT: medium chain triglycerides.

Sample		Raw	Cooked	∆E	∆C*	Mean Particle Diameter (µm)	Image
LCTs/Coarse	L*	62.0 ± 0.0 Aa	60.6 ± 0.5 Ab	8.9	8.8	9.86 ± 0.83	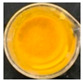
	a*	6.4 ± 0.0 Bb	7.8 ± 5.3 Aa				
	b*	29.6 ± 0.0 Ac	20.9 ± 0.5 Bd				
LCTs/Fine	L*	61.5 ± 0.4 Ba	65.6 ± 0.4 Aa	8.2	7.1	0.33 ± 0.03	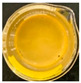
	a*	9.4 ± 0.6 Ba	7.0 ± 0.1 Aa				
	b*	46.9 ± 1.2 Ab	40.3 ± 0.6 Bb				
MCTs/Coarse	L*	33.0 ± 1.2 Bc	60.2 ± 0.3 Ab	28.3	7.7	12.5 ± 1.4	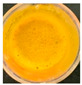
	a*	10.0 ± 0.4 Aa	3.7 ± 0.5 Bb				
	b*	29.6 ± 0.7 Ac	25.1 ± 0.8 Bc				
MCTs/Fine	L*	58.0 ± 0.2 Bb	62.9 ± 0.2 Aab	11.4	10.4	0.26 ± 0.01	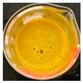
	a*	10.7 ± 0.1 Aa	6.8 ± 0.0 Ba				
	b*	58.1 ± 0.3 Aa	48.5 ± 0.2 Ba				

## Data Availability

The data that support the findings of this study are available from the corresponding author upon reasonable request.
